# The Tip of the Iceberg: Genotype of Puerto Rican Pediatric Obesity

**DOI:** 10.3390/genes15040394

**Published:** 2024-03-22

**Authors:** Jesus M. Melendez-Montañez, Wilfredo De Jesus-Rojas

**Affiliations:** Department of Pediatrics and Basic Science, Ponce Health Sciences University, Ponce, PR 00716, USA; jmelendez20@stu.psm.edu

**Keywords:** Puerto Rican genetics, obesity research, health studies, chronic disease, precision medicine

## Abstract

Childhood obesity is a significant public health concern, particularly among Hispanic populations. This study aimed to elucidate the genetic predisposition to obesity in Puerto Rican children of Hispanic descent, addressing a notable gap in existing research. A cohort of 103 children with obesity and hyperphagia underwent genetic screening for rare obesity-related variants. Clinical assessments and family history evaluations were conducted to characterize the demographic and clinical characteristics of the cohort. Genetic testing revealed a high prevalence of variants, with 73% of subjects having at least one reported variant. Pathogenic variants, predominantly associated with obesity-related ciliopathies, were identified in 7% of cases. Additionally, 90% of cases had variants of uncertain significance, highlighting the complexity of genetic contributions to obesity. This study emphasizes the critical need for further investigation into the genetic foundations of obesity, particularly within Hispanic communities. The findings emphasize the importance of early medical evaluation, vigilant monitoring for hyperphagia onset, and targeted interventions tailored to the unique genetic landscape of Puerto Rican children. This research provides a foundational framework for future studies to mitigate the impact of genetic obesity within this population.

## 1. Introduction

It is widely acknowledged that obesity is a multifaceted and intricate medical condition that is characterized by the excessive accumulation and dispersion of adipose tissue, which results in significant adverse effects on overall health and welfare [1,2]. Obesity is a prevalent issue in global public health [3]. It is possible to trace the origins of obesity back to the intricate interaction of genetic, environmental, and psychological factors, which, when taken together, manifest in a wide variety of phenotypic manifestations [4]. It is a matter of significant concern that the prevalence of overweight and obesity among children and adolescents is consistently on the rise [3,5]. Epidemiological data from the World Health Organization (WHO) characterize pediatric obesity as one of the most serious public health challenges of the 21st century, predisposing children to several health risks associated with the condition [6]. There exists a positive correlation between childhood overweight or obesity and the maintenance of obesity in adulthood, as well as an increased vulnerability to the onset of non-communicable diseases, including diabetes and cardiovascular diseases, during early stages of life [7]. In spite of the substantial amount of research that has been carried out on the physiological foundations of bodyweight management, the effectiveness of interventions that are aimed at addressing the issue of pediatric obesity is still primarily limited [8]. Prevention through promoting healthy diets, physical activity, and environments is crucial, as lifestyle modification post-obesity onset is challenging [9]. Behavioral and pharmacotherapy interventions show some success, but further research into effective prevention and treatment methods is needed [9]. Continued research is needed to understand genetic and biological factors influencing weight gain and therapeutic responses [9].

The prevalence of obesity can be attributed to a combination of dietary and behavioral patterns that influence an individual’s genetic composition, ultimately determining their body mass and vulnerability to diseases associated with obesity [10]. The identification of obesity-related genes has been facilitated by recent advancements in genetic evaluation and analysis. For instance, a total of eight genes have been identified as potential contributors to obesity. These genes include leptin (*LEP*), the leptin receptor (*LEPR*), proopiomelanocortin (*POMC*), prohormone convertase 1 (*PCSK1*), the melanocortin 4 receptor (*MC4R*), single-minded homolog 1 (*SIM1*), brain-derived neurotrophic factor (*BDNF*), and the neurotrophic tyrosine kinase receptor type 2 gene (*NTRK2*). These genes have been identified from a total of over 500 genes associated with obesity [11,12]. Obesity commonly exhibits a significant hereditary element, although the specific genetic mechanisms that contribute to obesity remain unclear [13]. Numerous genetic association studies have been documented. However, only a limited number have been effectively reproduced [4]. Advancements in research methodologies and extensive investigations will facilitate comprehension of genetic factors and their interplay in the development of obesity, thereby informing effective interventions and therapies [14]. Obesity is a multifaceted condition that is affected by various genes [15]. The heritability of obesity-related phenotypes in humans is estimated to range from 40 to 70 percent [4]. Recent studies indicate that genetics exert a substantial influence on an individual’s susceptibility to obesity [16]. The body’s ability to regulate energy balance and control weight is influenced by the interplay between genes and the environment [4]. Advancements in genetic research have led to the identification of numerous genes that influence obesity, particularly in cases of early-onset severe obesity [4]. The genetic factors contributing to obesity have yielded significant knowledge regarding the fundamental mechanisms and pathways implicated in its development [4,11,17]. Comprehending the genetic underpinnings of obesity has facilitated the exploration of probable therapeutic alternatives and interventions aimed at particular genetic variants linked to obesity [16]. Obesity is a multifaceted and inheritable characteristic impacted by various factors, including genetics, epigenetics, metagenomics, and the environment [18].

Over the last few decades, Puerto Rico has had an alarmingly higher rate of pediatric obesity compared to the mainland U.S. [19,20]. Several studies have presented evidence indicating that Puerto Rican children are disproportionately affected by obesity, with higher rates of occurrence compared to children from other ethnic backgrounds [21]. The elevated occurrence of childhood obesity among Puerto Ricans has been attributed to a distinct combination of genetic predisposition and the cultural and social milieu, suggesting that this distinctive interaction may explain the observed phenomenon Nevertheless, there has been a lack of in-depth research regarding the participation of obesity-associated genes in Puerto Rican children. A multitude of genes have been associated with both rare syndromic and non-syndromic genetic disorders that are associated with severe obesity, such as Bardet-Biedl syndrome, Alström syndrome, and Prader-Willi syndrome. All of these disorders share a common factor that acts as a mediator, specifically hyperphagia, which is the involuntary desire to consume food [22]. Understanding the genetics of hunger and satiety through different disease mechanisms has led to the idea that it could provide evidence about the connection between obesity, genes, and diseases [23]. The pathogenesis of obesity involves various mechanisms, including the leptin-melanocortin signaling pathway, genes related to energy homeostasis in neuron development, and syndromic obesity associated with ciliopathies [24]. However, there has been a lack of research on the connections and mechanisms between pediatric obesity, genes, and diseases in Puerto Rico. The aim of the study is to characterize the genotypic-phenotypic aspects of obesity in the Pediatric Puerto Rican population to uncover the “tip of the iceberg” of a multifaceted and complex disease like obesity.

## 2. Materials and Methods

### 2.1. Study Design and Participants

We conducted an exploratory study on 103 children evaluated for rare obesity disorders through genetic screening at a single outpatient pediatric clinic. This retrospective study included patients referred to the Rare Lung and Pediatric Asthma Institute in Puerto Rico, diagnosed with asthma, obstructive sleep apnea, and/or obesity between January 2022 and July 2023. The retrospective nature of our study, which relied on pre-existing data, did not necessitate obtaining written consent, as per the guidelines set forth by our Institutional Review Board. Inclusion criteria were children aged ≤ 18 years with a BMI ≥ 97th percentile for age and sex.

### 2.2. Clinical Assessment

During the clinical encounter, a thorough assessment was conducted, encompassing an in-depth examination of the patient’s family history, measurement of weight, height, and body mass index (BMI). The assessment of hyperphagia involved conducting parental interviews that specifically examined the child’s eating habits, the frequency of hunger sensations, and the level of satiety experienced after meals or snacks.

### 2.3. Obesity Genetic Screening

Taking into consideration the child’s history of obesity, patterns of weight gain, eating concerns, clinical suspition of syndromic obesity, or history of obesity in the child’s parents, genetic testing was strongly considered. Through a program that was sponsored by Rhythm Pharmaceuticals in Boston, Massachusetts, United States of America, buccal swab samples were collected for the purpose of genetic analysis. This analysis was carried out at no cost to the participants. For the purpose of processing, samples were delivered to Rhythm Pharmaceuticals Laboratories, which is located in Marshfield, Wisconsin, in the United States.

### 2.4. Genetic Panel and Analysis

**Figure 1:**
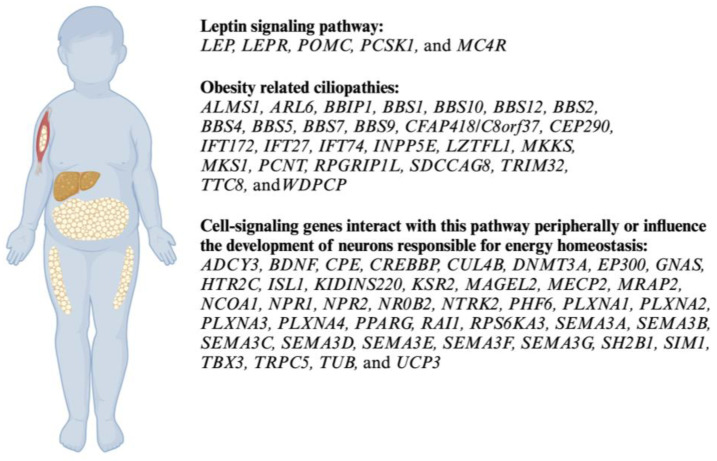
Obesity mechanism per group of genes analyzed. Created with BioRender.com.

The genetic panel targeted 79 genes associated with non-syndromic obesity, syndromic obesity, Bardet–Biedl syndrome, and related ciliopathies. These genes are involved in the leptin-melanocortin signaling pathway, ciliopathies, peripheral cell signalig and/or the development of neurons responsible for energy homeostasis (Figure 1). The panel achieved 99.92% coverage of all coding exons, plus 10 bases of flanking noncoding DNA, and included other non-coding regions where pathogenic variants have been identified. PreventionGenetics defined coverage as ≥20× NGS reads or Sanger sequencing.

### 2.5. Reports and Counseling

The genetic testing reports provided detailed information on the identified gene(s), including the OMIM number, mode of inheritance, genetic variant details, ClinVar ID, allele frequency in gnomAD, in silico missense predictions, and variant interpretation. Interpretations followed the American College of Medical Genetics guidelines [25] categorizing gene variants as pathogenic, likely pathogenic, variant of uncertain significance (VUS), or at risk for obesity. Only pathogenic, likely pathogenic, and VUS findings were reported to families, who were then offered genetic counseling by Rhythm Pharmaceuticals or referred to services with local geneticists.

### 2.6. Statistical Analysis

The study provided descriptive data for categorical variables, including child sex, age group, race, and ethnicity, both overall and by gene variant group. The study provided descriptive statistics in the form of medians, interquartile ranges, and percentages. On 23 February 2024, statistical analysis was performed using GraphPad Prism version 10.2.0 for iOS (GraphPad Software, San Diego, CA, USA, www.graphpad.com) (accessed on 1 February 2024).

## 3. Results

### 3.1. Demographics and Clinical Characteristics

A cohort consisting of 103 children who exhibited both obesity and hyperphagia at a single outpatient pediatric clinic underwent screening to identify uncommon genetic factors contributing to obesity. There was no evidence of siblings or consanguinity among the children in the group. Among the group, 35% (36/103) were female, and all of them were of Hispanic descent. The median age of the children was nine years old (IQR, 6). The median BMI (kg/m^2^) for females was 31.7 (IQR, 12.8), and for males was 29.4 (IQR, 7.5) (Table 1). A total of 87 patients (84%) reported experiencing hyperphagia, with onset occurring at a median age of four years old (IQR, 2) for females and five years old (IQR, 2.5) for males. Most (93/103, 90%) of the children had a family history of obesity, as confirmed through medical evaluation (Table 1).

### 3.2. Genetic Test Results

The genetic results of our 103 subjects were positive (1/103, 1%), indeterminate in (76/103, 73%), and negative in (27/103, 26%) in genetic tests. From a total of 157 variants identified, 99 were autosomal recessive (AR) (63%), 29 were autosomal dominant (AD) (18%), seven were AR and AD (5%), and 21 were of unknown mode of inheritance (14%). The median gnomAD allelic frequency (%) of 138 variants was 0.03 (IQR, 0.05), and in 15 variants (11%), gnomAD allelic frequency was not present in the population. The three most common gnomAD population frequencies in the variants of our pediatric cohort were Latino (34.8%), European (non-Finnish) (24.6%), and African (21.7%) (Table 2).

#### 3.2.1. Overview of Obesity Genetic Variants

Seventy-six subjects (73% of individuals tested) had at least one variant reported among the analyzed genes; 30 (39%) had a single variant, and 46 (61%) reported more than one variant. Twenty-two youths had two variants, 16 youths had three variants, five had four variants, and three had five variants reported (Table S1). One variant, c.2494T>C in *PLNXNA2*, was hemizygous; all other variants found were heterozygous with no complex heterozygosity (Table S1). Among subjects with a variant identified, 157 unique variants were reported in 46 of the 79 genes analyzed from the genetic panel (Table S2). Among those screened, variants were found almost equally among all age groups (Table S1).

#### 3.2.2. Pathogenic, Likely Pathogenic, or At-Risk Variants

Eleven pathogenic variants (7%) were identified in our cohort of 157 variants. The four most frequent pathogenic variants, present at least two or more times in our cohort, were c.661A>G on *PCSK1*, c.1169T>G on *BBS1*, c.1645G>T on *BBS1*, and c.1421C>T on *TUB* (Table 3). Other seven pathogenic variants were present, such as c.152G>C on *BBS12*, c.1530G>A on *MKKS*, c.1963C>A on *SEMA3F*, c.2365A>G on *BBS9*, c.375G>A on *PCSK1*, c.396G>C on *BBS9*, and c.9752dup on *PCNT*. Most of the genes involved in the pathogenic variants were linked to obesity-related ciliopathies (7/11, 64%), followed by the leptin signaling pathway (2/11, 18%) and cell-signaling regulation genes (2/11, 18%) (Figure 1).

#### 3.2.3. Variants of Uncertain Significance (VUS)

A total of 141 VUS (90%) in our cohort were identified. The nine most frequent VUS, present at least three or more times in our cohort, were c.396G>C on *BBS9*, c.1028C>T on *SH2B1*, c.1918A>G on PCSK1, c.1963C>A on *SEMA3F*, c.251G>A on *RPGRIP1L*, c.5528A>T on *VPS13B*, c.833G>A on *SDCCAG8*, c.83T>C on *MKS1*, and c.857A>G on *MKS1* (Table 3). Most of the genes involved in the VUS were obesity-related ciliopathies (16/28, 57%), followed by the cell-signaling regulation genes (6/28, 21%), leptin signaling pathway (3/28, 11%), and unknown mechanisms (3/28, 11%) (Figure 1).

## 4. Discussion

The unique nature of our study lies in its exclusive focus on Puerto Rican children of Hispanic heritage. Prior studies investigating the etiology of childhood obesity have predominantly concentrated on diverse demographic groups, wherein Hispanics have constituted a relatively minor fraction of the overall sample. [26]. Our demographic findings, combined with those from other studies, suggest that Hispanics may have a genetic predisposition to obesity [26,27]. Additional investigation is required to gain a more comprehensive understanding of the correlation between genetics and obesity in different Hispanic populations [28]. To our knowledge, this is the first study in Puerto Rico that explores the genetics of obesity in children. This study conducted a retrospective analysis of 103 pediatric patients, a sample size that appears to be sufficient in comparison to previous studies [26,29]. None of the subjects studied were siblings or had consanguinity among families; comparable studies did not investigate consanguinity or found that some subjects were related [26,27,29]. Our cohort had a BMI ≥ 97th percentile of roughly 99% and 84% hyperphagia, similarly seen in Roberts et al.’s [26]. In our study, females experienced hyperphagia at a median age of four years, while males experienced it at the age of five. This finding underscores the significance of conducting a comprehensive medical assessment and highlights the potential benefits of creating a validated hyperphagia questionnaire for the diagnosis of pediatric genetic obesity, a symptom that is typically underexplored in the field of pediatrics [30]. Consequently, it is imperative for pediatricians in Puerto Rico to initiate the surveillance of hyperphagia during the early stages of childhood. In both previous studies, a family history of obesity was assessed, with rates of at least 90% [26,27]. The study cohort exhibited a similar trend, as 90% of the participants had a familial predisposition to obesity. The assessment of genetic obesity in pediatric patients requires the examination of clinical characteristics, including body mass index (BMI) at the 97th percentile, hyperphagia, and familial predisposition to obesity [31]. Additional publications have corroborated the evidence regarding the utilization of genetic testing in the assessment of obese patients [31,32,33]. These publications stress the need for precision medicine and the creation of personalized treatments for genetic variants that cause rare genetic obesity [31,32,33].

The genetic testing conducted in our study produced 1% positive results, 73% indeterminate results, and 27% negative results. In comparison to other studies, this finding is comparable [26,29]. Furthermore, the group of tests in similar studies exhibited a comparable distribution of favorable outcomes, despite 38.5% producing unfavorable results. The exact percentage of uncertain results was not explicitly stated [26,29]. The presence of indeterminate genetic results within our cohort underscores the necessity for additional research endeavors aimed at enhancing comprehension and establishing correlations between genetic variants associated with obesity and the clinical manifestations of genetic obesity. Due to the limited genetic investigation into obesity within the Puerto Rican population, it is imperative to prioritize the examination of VUS as a potential genetic determinant of obesity. The mode of inheritance observed in our cohort was primarily AR (63%), followed by AD (5%), and notably, 14% exhibited an unknown mode of inheritance. In a study that was similar to ours, the majority of these findings were less common, but the modes of inheritance for AD and AR were similar [26]. Suggests that these findings could be due to randomness and not to any specific factor. Similarly, a median gnomAD allelic frequency of 0.03%, with 11% of the variants not present in gnomAD. In the gnomAD database, our findings diverged from those of other studies, revealing a higher prevalence of variants associated with Latinos (34.8%) yet displaying comparable frequencies in African and European (non-Finnish) populations. [2]. These findings were expected because our cohort was entirely Hispanic and Puerto Rican. In Roberts et al.’s study, populations such as South Asian, Ashkenazi Jewish, East Asian, and European (Finnish) were more frequent in their cohort [26]. This reiterates that our results largely depend on the population examined; thus, further research is necessary to determine whether this trend persists.

Among the children tested in our study, 73% had at least one variant reported, 61% had more than one variant, and 39% had one variant. Other studies showed similar frequencies, such as 61.5%, 69.4%, and 30.1% [26,29]. Also, variants in our study and the other studies were found equally among all age groups [26,29]. The frequency of pathogenic variants in our cohort of 157 variants was 7%, similar to other rare obesity studies [26,29,34]. Elevated frequencies were seen in Roberts et al.’s 2022 study, depicting nine pathogenic variants, specifically the c.661A>G variant of *PCSK1* [26]. As a result, *PCSK1* pathogenic variants may need to be explored further to determine clinical implications. Another finding in our study cohort was an elevated frequency of *BBS1* pathogenic variants. Similar frequencies of pathogenic variants were seen in a study looking at the importance of genetic testing in obesity [29]. The rare pathogenic variant c.1421C>T in *TUB* present in our study was not reported in other studies [26,29,34]. Further studies are needed to correlate these variants with clinical manifestations in obese pediatric patients. 

The gene variants seen in our cohort were associated with three diseases: hyperphagic obesity with impaired prohormone processing, Bardet–Biedl syndrome 1 (BBS1), and human obesity. Studies have shown that BBS1 is associated with rapid weight gain and early hyperphagia onset [35]. However, these findings worsened after subjects were older than 11 years old [35]. These findings correlate with our demographic and clinical characteristics data. This suggests that *BBS1* pathogenic variants such as c.1645G>T could be involved in the pathogenesis of obesity in our cohort, and more studies into this variant in Puerto Rico are needed. Guardiola et al.’s study that revealed that the c.1645G>T (p.Glu549*) mutation in Puerto Rico exhibits more pronounced characteristics of BBS1, as opposed to the less severe features observed in other mutations that cause the syndrome [36]. Similarly, studies on *PCSK1* have shown that specific mutations lead to a deficiency in proinsulin processing, resulting in hyperphagic obesity [37]. Therefore, these findings could suggest that the pathogenic variant c.661A>G on *PCSK1* is related to obesity in our cohort. Finally, a prior study established a clear relationship between *TUB* and obesity by examining its expression in the hypothalamus and adipose tissue [38]. In addition, it showed how *TUB* could be a potential biomarker or therapeutic target for obesity-related conditions [38]. Considering the number of subjects with the pathogenic variant *TUB* and the clinical characteristics of obesity observed, we could hypothesize that it causes severe obesity in children. Obesity-related ciliopathies were the most common mechanism based on positive genes for obesity (64%). These were followed by the leptin signaling pathway (18%) and the cell signaling pathway (18%). This trend is also seen in similar studies [26,29,34,39]. This emphasizes the need for additional research on ciliopathies, which have shown a higher prevalence in our study and others, to advance the development of targeted treatments and clarify the precise pathophysiology of obesity.

Our cohort identified 141 VUS, accounting for 90% of the cases. These findings echo similar observations in similar studies, emphasizing the necessity for further investigation into these variants and their implications in rare genetic obesity [26,29,34]. Among the VUS identified, nine variants were notably recurrent, occurring at least three times each within our cohort. The Roberts et al.’s study noted a comparable incidence of other VUS [26]. Most genes linked to the identified VUS are connected to ciliopathies linked to obesity (57%). These are followed by genes that control cell signaling (21%), the leptin signaling pathway (11%), and genes whose mechanisms are unknown (11%), such as *VPS13B*. This distribution aligns with the findings reported in similar studies [26,29,34]. These findings highlight the importance of investigating the pathological mechanisms behind these variants of obesity and examining how VUS could impact obesity in the Puerto Rican pediatric population as more genetic data becomes available. 

Our study has a few limitations, including a sample comprising 103 children recruited from a single outpatient clinic. A limited and geographically concentrated sample size may restrict the extrapolation of our results to the broader population of Puerto Rican children or other Hispanic communities. However, it provides valuable insights into the genetics of obesity within this specific group. The retrospective nature of our study, coupled with its reliance on medical records for data collection, could predispose our findings to recall bias or incomplete data, especially concerning detailed family histories and clinical evaluations. Although these limitations may affect the precision and depth of our analysis, they also highlight the importance of utilizing retrospective data to identify trends and potential genetic markers of obesity in specific populations like Puerto Ricans. Notably, identifying VUS in 90% of our cases poses a substantial challenge for clinical interpretation, highlighting the need for further functional studies to clarify their role in obesity within under-researched populations such as the Puerto Rican community. The ethnic homogeneity of our cohort, while beneficial for reducing genetic variability, may also constrain our understanding of the broader genetic diversity and its implications for obesity across different Hispanic populations. This limitation underscores the importance of conducting future research with larger, more ethnically diverse cohorts, employing prospective study designs, and incorporating comprehensive functional analyses. Such efforts are essential to advance our understanding of genetic obesity in Puerto Rican children and to extend these insights to other Hispanic communities, ultimately facilitating the development of targeted therapeutic interventions and preventive strategies in the future.

## 5. Conclusions

In conclusion, our research, which centers on Puerto Rican children of Hispanic heritage, offers a more comprehensive understanding of the genetic inclination towards obesity. By acknowledging a notable limitation in current scientific research that predominantly focuses on other demographic groups, we underscore the significance of conducting further research into genetic obesity, specifically within Hispanic communities. The need of early medical evaluation and vigilant monitoring for the onset of hyperphagia can be seen by our findings. Additionally, we highlight the potential importance of screening for genetic obesity through comprehensive clinical assessments and consideration of familial history. The high occurrence of VUS, specifically those linked to ciliopathies, highlights the complex nature of genetic obesity and underscores the continuous requirement for thorough research to understand its intricacies. Our study has the potential to reveal only a small portion of the problem, which is a significant advancement in our knowledge of genetic obesity among the Puerto Rican population.

## Figures and Tables

**Table 1:** Demographics and clinical characteristics.

Characteristics	Value (*n* = 103)
Gender (F/M)	36/67
Age, median (IQR), (years)	9 (6)
Ethnicity, *n* (%), Hispanics, Puerto Ricans	103 (100)
BMI females, median (IQR), (kg/m^2^)	31.7 (12.8)
BMI males, median (IQR), (kg/m^2^)	29.4 (7.5)
Hyperphagia	87 (84)
Age onset of hyperphagia females, median (IQR), (years)	4 (2)
Age of onset of hyperphagia males, median (IQR), (years)	5 (2.5)
Family history of obesity, *n* (%)	93 (90)

F: Female, M: male, IQR: interquartile range.

**Table 2:** Most common gnomAD population frequencies in our study cohort.

Population	Frequency (*n* = 138)
Latino (*n*, %)	(48, 34.8)
European (non-Finnish)	(34, 24.6)
African	(30, 21.7)
South Asian	(12, 8.7)
Ashkenazi Jewish	(10, 7.2)
East Asian	(2, 1.4)
European (Finnish)	(2, 1.4)

A total of 19 genetic variants from our cohort (*n* = 157) were not listed.

**Table 3:** Variants identified within the pediatric population with obesity in Puerto Rico.

Gene	Variant	Frequency	Classification
*PCSK1*	c.661A>G	5	Pathogenic
*BBS1*	c.1169T>G	4	Pathogenic
*BBS1*	c.1645G>T	3	Pathogenic
*TUB*	c.1421C>T	2	Risk
*BBS9*	c.396G>C	4	VUS
*SH2B1*	c.1028C>T	3	VUS
*PCSK1*	c.1918A>G	3	VUS
*SEMA3F*	c.1963C>A	3	VUS
*RPGRIP1L*	c.251G>A	3	VUS
*VPS13B*	c.5528A>T	3	VUS
*SDCCAG8*	c.833G>A	3	VUS
*MKS1*	c.83T>C	3	VUS
*MKS1*	c.857A>G	3	VUS

*BBS9*: Bardet–Biedl Syndrome 9, *SH2B1*: SH2B Adaptor Protein 1, *PCSK1*: Proprotein Convertase Subtilisin/Kexin Type 1, *SEMA3F*: Semaphorin 3F, *RPGRIP1L*: RPGRIP1 Like, *VPS13B*: Vacuolar Protein Sorting 13 Homolog B, *SDCCAG8*: Serologically Defined Colon Cancer Antigen 8, *MKS1*: Meckel Syndrome 1. *BBS1*: Bardet–Biedl Syndrome 1, *TUB*: Tubby Bipartite Transcription Factor. VUS: Variant of uncertain significance.

## Data Availability

All data are available upon request through the corresponding author.

## Supplementary Materials

The following supporting information can be downloaded at: https://www.mdpi.com/article/10.3390/genes15040394/s1, Table S1: Variant analysis from pediatric Puerto Rican obesity cohort; Table S2: Genes analyzed by Rhythm Pharmaceuticals Laboratories (Prevention Genetics, Marshfield, WI, USA).
