# Supplementary material for: The Tip of the Iceberg: Genotype of Puerto Rican Pediatric Obesity
Source: Genes (Basel). 2024 Mar 22;15(4):394. doi: 10.3390/genes15040394 (PMC11049160; doi:10.3390/genes15040394)
Supplement: Supplementary file 1 [file genes-15-00394-s001.zip › Genes Analyzed table S2.pdf]

Gene	Gene Name	NCBI Accession Number
ADCY3	Adenylate Cyclase 3	NC_000001.11
AFF4	AF4/FMR2 Family Member 4	NC_000005.10
ALMS1	Alström Syndrome 1	NC_000002.12
ARL6	ADP Ribosylation Factor Like GTPase 6	NC_000003.12
BBIP1	BBSome Interacting Protein 1	NC_000012.12
BBS1	Bardet-Biedl Syndrome 1	NC_000011.10
BBS10	Bardet-Biedl Syndrome 10	NC_000012.12
BBS12	Bardet-Biedl Syndrome 12	NC_000004.12
BBS2	Bardet-Biedl Syndrome 2	NC_000016.10
BBS4	Bardet-Biedl Syndrome 4	NC_000015.10
BBS5	Bardet-Biedl Syndrome 5	NC_000002.12
BBS7	Bardet-Biedl Syndrome 7	NC_000004.12
BBS9	Bardet-Biedl Syndrome 9	NC_000007.14
BDNF	Brain-Derived Neurotrophic Factor	NC_000011.10
CEP290	Centrosomal Protein 290	NC_000012.12
CFAP418	Cilia And Flagella Associated Protein 418	NC_000004.12
CPE	Carboxypeptidase E	NC_000004.12
CREBBP	CREB Binding Protein	NC_000016.10
CUL4B	Cullin 4B	NC_000023.11
DNMT3A	DNA Methyltransferase 3 Alpha	NC_000002.12
DYRK1B	Dual-Specificity Tyrosine-(Y)-Phosphorylation Regulated Kinase 1B	NC_000019.10
EP300	E1A Binding Protein P300	NC_000022.11
GNAS	GNAS Complex Locus	NC_000020.11
HTR2C	5-Hydroxytryptamine Receptor 2C	NC_000023.11
IFT172	Intraflagellar Transport 172	NC_000008.11
IFT27	Intraflagellar Transport 27	NC_000001.11
IFT74	Intraflagellar Transport 74	NC_000022.11
INPP5E	Inositol Polyphosphate-5-Phosphatase E	NC_000009.12
ISL1	ISL LIM Homeobox 1	NC_000005.10
KIDINS220	Kinase D Interacting Substrate 220	NC_000018.10
KSR2	Kinase Suppressors of Ras 2	NC_000012.12
LEP	Leptin	NC_000016.10
LEPR	Leptin Receptor	NC_000001.11
LZTFL1	Leucine Zipper Transcription Factor Like 1	NC_000003.12
MAGEL2	MAGE Family Member L2	NC_000015.10
MC3R	Melanocortin 3 Receptor	NC_000020.11
MC4R	Melanocortin 4 Receptor	NC_000018.10
MECP2	Methyl-CpG Binding Protein 2	NC_000023.11
MKKS	McKusick-Kaufman Syndrome	NC_000020.11
MKS1	Meckel Syndrome 1	NC_000017.11
MRAP2	Melanocortin 2 Receptor Accessory Protein 2	NC_000019.10
NCOA1	Nuclear Receptor Coactivator 1	NC_000002.12
NR0B2	Nuclear Receptor Subfamily 0 Group B Member 2	NC_000001.11
NRP1	Neuropilin 1	NC_000010.11
NRP2	Neuropilin 2	NC_000002.12
NTRK2	Neurotrophic Tyrosine Kinase Receptor Type 2	NC_000009.12
PCNT	Pericentrin	NC_000021.9
PCSK1	Proprotein Convertase Subtilisin/Kexin Type 1	NC_000005.10
PHF6	PHD Finger Protein 6	NC_000007.14
PHIP	Pleckstrin Homology Domain Interacting Protein	NC_000006.12
PLXNA1	Plexin A1	NC_000003.12
PLXNA2	Plexin A2	NC_000001.11
PLXNA3	Plexin A3	NC_000003.12
PLXNA4	Plexin A4	NC_000012.12
POMC	Proopiomelanocortin	NC_000002.12
PPARG	Peroxisome Proliferator Activated Receptor Gamma	NC_000003.12
PROK2	Prokineticin 2	NC_000003.12
RAB23	RAB23, Member RAS Oncogene Family	NC_000006.12

RAI1	Retinoic Acid Induced 1	NC_000017.11
RPGRIP1L	RPGRIP1 Like	NC_000014.9
RPS6KA3	Ribosomal Protein S6 Kinase A3	NC_000006.12
SDCCAG8	Serologically Defined Colon Cancer Antigen 8	NC_000002.12
SEMA3A	Semaphorin 3A	NC_000005.10
SEMA3F	Semaphorin 3F	NC_000003.12
SEMA3G	Semaphorin 3G	NC_000003.12
SH2B1	SH2B Adaptor Protein 1	NC_000016.10
SIM1	SIM BHLH Transcription Factor 1	NC_000006.12
TUB	Tubby Bipartite Transcription Factor	NC_000007.14
UCP3	Uncoupling Protein 3	NC_000011.10
VPS13B	Vacuolar Protein Sorting 13 Homolog B	NC_000008.11

Table S2: Genes analyzed by Rhythm Pharmaceuticals Laboratories (PreventionGenetics, Marshfield, WI, USA).
