# Supplementary material for: The Tip of the Iceberg: Genotype of Puerto Rican Pediatric Obesity
Source: Genes (Basel). 2024 Mar 22;15(4):394. doi: 10.3390/genes15040394 (PMC11049160; doi:10.3390/genes15040394)
Supplement: Supplementary file 1 [file genes-15-00394-s001.zip › Table S1.pdf]

IDN	Age	Gene	DNA Variant	Predicted protein change	Mode of Inheritance	Zygocity	ClinVar ID	Highest Allele Frequency in a gnomAD population (%)	In Silico Missense Predictions	Interpretation	Disease associated
**1**	9	*ALMS1*	c.3907_3909del	p.Ser1303del	AR	Heterozygous	550818	0.0035	Not applicable	VUS	Alstrom syndrome
**1**	9	*MKS1*	c.322C>T	p.Arg108Cys	AR	Heterozygous	853823	0.059	Conflicting	VUS	Bardet-Biedl syndrome 13 OMIM #615990, autosomal recessive Joubert syndrome 28 OMIM #617121, and autosomal recessive Meckel-Gruber syndrome 1 OMIM #249000
**2**	11	*ALMS1*	c.12001C>T	p.Arg4001Trp	AR	Heterozygous	391899	0.05	Conflicting	VUS	Alstrom syndrome
**2**	11	*ALMS1*	c.5459C>T	p.Pro1820Leu	AR	Heterozygous	241003	0.091	Conflicting	VUS	Alstrom syndrome
**2**	11	*BBS9*	c.2365A>G	p.Lys789Glu	AR	Heterozygous	971120	0.00088	Conflicting	VUS	Bardet-Biedl syndrome 9
**3**	9	*MKS1*	c.199C>T	p.Arg67Cys	AR	Heterozygous	461763	0.087	Not available	VUS	Bardet-Biedl syndrome 13
**3**	*9*	*TUB*	c.161C>A	p.Thr54Lys	AR	Heterozygous	1006177	0.087	Not available	VUS	retinal dystrophy and obesity
**5**	*7*	*BBS1*	c.1169T>G	p.Met390Arg	AR	Heterozygous	12143	0.28	Not available	Pathogenic	Bardet-Biedl syndrome 1
**5**	*7*	*BBS12*	c.152G>C	p.Ser51Thr	AR	Heterozygous	968505	0.0087	Not available	VUS	Bardet-Biedl syndrome 12
**5**	*7*	*RAI1*	c.2422G>A	p.Gly808Arg	AR	Heterozygous	196538	0.026	Not available	VUS	Smith-Magenis syndrome
**7**	10	*LEPR*	c.296G>A	p.Cys99Tyr	Not Listed	Not listed	.	0.038	Conflicting	VUS	obesity and hypogonadotropic hypogonadism
**7**	*10*	*POMC*	c.706C>G	p.Arg236Gly	AR, AD	Heterozygous	13356	0.78	Damaging	VUS	severe obesity
**8**	15	*BBS1*	c.1645G>T	p.Glu549*	AR	Heterozygous	12144	0.0087	Not available	Pathogenic	Bardet-Biedl syndrome 1
**9**	6	*MKS1*	c.857A>G	p.Asp286Gly	AR	Heterozygous	609883	0.099	Conflicting	VUS	Bardet-Biedl syndrome 13
**9**	*6*	*MKS1*	c.83T>C	p.Val28Ala	AR	Heterozygous	609883	0.007	Conflicting	VUS	Bardet-Biedl syndrome 13
**10**	18	*PCNT*	c.9800G>A	p.Arg3267His	AR	Heterozygous	Not listed	0.02	Tolerated	VUS	microcephalic osteodysplastic primordial dwarfism, type II.
**10**	*18*	*SEMA3F*	c.1963C>A	p.Arg655Ser	Unknown	Heterozygous	Not listed	0.058	Conflicting	VUS	obesity
**11**	16	*PLXNA2*	c.614G>A	p.Arg205Gln	Unknown	Heterozygous	Not listed	0.068	Conflicting	VUS	autism spectrum disorder
**11**	*16*	*SH2B1*	c.1028C>T	p.Thr343Met	AR	Heterozygous	Not listed	0.048	Conflicting	VUS	obesity, insulin resistance and maladaptive behavior phenotypes
**11**	*16*	*TUB*	c.901G>A	p.Val301Ile	AR	Heterozygous	956470	0.015	Conflicting	VUS	recessive retinal dystrophy and obesity
**12**	9	*MAGEL2*	c.1266_1286dup	p.Ile423_Pro429dup	AR	Heterozygous	Not listed	0.014	.	VUS	Schaaf-Yang syndrome
**12**	*9*	*PCSK1*	c.661A>G	p.Asn221Asp	AR	Heterozygous	14040	6.6	Conflicting	RISK	obesity with impaired prohormone processing
**14**	*7*	*SH2B1*	c.1028C>T	p.Thr343Met	AR	Heterozygous	Not listed	0.048	Conflicting	VUS	obesity, insulin resistance and maladaptive behavior phenotypes
**16**	5	*PCSK1*	c.661A>G	p.Asn221Asp	AR	Heterozygous	14040	6.6	Confliction	RISK	obesity with impaired prohormone processing
**17**	18	*BBS9*	c.396G>C	p.Gln132His	AR	Heterozygous	841915	0.042	Damaging	VUS	Bardet-Biedl syndrome 9
**17**	*18*	*LEPR*	c.658G>A	p.Val220Ile	AR	Heterozygous	917428	0.13	Tolerated	VUS	obesity and hypogonadotropic hypogonadism due to leptin receptor deficiency
**18**	4	*PCNT*	c.9800G>A	p.Arg3267His	AR	Heterozygous	Not listed	0.02	Tolerated	VUS	autosomal recessive microcephalic osteodysplastic primordial dwarfism, type II
**18**	*4*	*PCSK1*	c.1918A>G	p.Thr640Ala	AR	Heterozygous	436278	0.45	Tolerated	VUS	obesity with impaired prohormone processing
**21**	11	*ADCY3*	c.1222G>A	p.Gly408Arg	AR	Heterozygous	Not listed	0.029	Conflicting	VUS	autosomal recessive severe obesity
**21**	*11*	*PLXNA1*	c.224C>T	p.Ser75Leu	Unknown	Heterozygous	Not listed	0.0062	Conflicting	VUS	Semaphorin 3 signaling and may be associated with obesity
**21**	*11*	*SEMA3F*	c.229G>A	p.Val77Met	Unknown	Heterozygous	Not listed	0.09	Conflicting	VUS	obesity
**22**	13	*LEPR*	c.430G>T	p.Val144Leu	AR	Heterozygous	393362	0.024	Tolerated	VUS	obesity and hypogonadotropic hypogonadism due to leptin receptor deficiency
**24**	14	*VPS13B*	c.4254T>G	p.His1418Gln	AR	Heterozygous	626047	0.0058	Conflicting	VUS	.
**26**	15	*VPS13B*	c.559C>T	p.Arg187Cys	AR	Heterozygous	570075	0.0035	Damaging	VUS	.
**27**	8	*PLXNA1*	c.1825G>A	p.Val609Ile	Unknown	Heterozygous	Not listed	0.02	Tolerated	VUS	developmental delay and autism spectrum disorder
**27**	*8*	*PLXNA1*	c.2618C>T	p.Thr873Met	Unknown	Heterozygous	Not listed	0.0085	Conflicting	VUS	developmental delay and autism spectrum disorder
**27**	*8*	*RAI1*	c.5435C>T	p.Pro1812Leu	AR	Heterozygous	1204491	0.039	Conflicting	VUS	Smith-Magenis syndrome
**29**	2	*IFT172*	c.886C>T	p.Arg296Trp	AR	Heterozygous	97028	0.11	Damaging	VUS	Mainzer-Saldino syndrome

**29**	2	*NRP2*	c.2681A>C	p.Glu894Ala	AR	Heterozygous	Not listed	.	Damaging	VUS	Semaphorin 3 signaling and may be associated with obesity
**29**	2	*UCP3*	c.58G>A	p.Ala20Thr	AR	Heterozygous	Not listed	0.029	Damaging	VUS	severe obesity
**38**	17	*VPS13B*	c.5528A>T	p.Glu1843Val	AR	Heterozygous	966951	0.032	Conflicting	VUS	Cohen syndrome
**38**	17	*KIDINS220*	c.136G>A	p.Ala46Thr	AR, AD	Heterozygous	1365824	0.12	Conflicting	VUS	de novo in patients with spastic paraplegia, intellectual disability, nystagmus, and obesity
**44**	6	*PCNT*	c.9752dup	p.Thr3252Asnfs*84	AR	Heterozygous	Not listed	0.012	Not applicable	Likely Pathogenic	autosomal recessive microcephalic osteodysplastic primordial dwarfism, type II
**44**	6	*PLXNA3*	c.1709C>T	p.Ala570Val	Unknown	Heterozygous	Not listed	0.027	Tolerated	VUS	obesity, mild intellectual disability, and autism spectrum disorder
**49**	11	*BBS9*	c.2593A>G	p.Thr865Ala	AR	Heterozygous	360072	0.051	Tolerated	VUS	Bardet-Biedl syndrome 9
**49**	11	*EP300*	c.1991T>C	p.Met664Thr	AR	Heterozygous	1201502	0.0096	Conflicting	VUS	Autosomal Dominant Rubinstein-Taybi syndrome 2, and Menke-Hennekam syndrome 2
**49**	11	*KIDINS220*	c.3548C>G	p.Ala1183Gly	AR, AD	Heterozygous	Not listed	0.017	Conflicting	VUS	de novo in patients with spastic paraplegia, intellectual disability, nystagmus, and obesity
**50**	8	*NRP2*	c.1490C>T	p.Thr497Ile	Unknown	Heterozygous	Not listed	0.00088	Conflicting	VUS	Semaphorin 3 signaling and may be associated with obesity
**50**	8	*VPS13B*	c.4254T>G	p.His1418Gln	AR	Heterozygous	626047	0.0058	Conflicting	VUS	Cohen syndrome
**53**	13	*EP300*	c.2645C>G	p.Pro882Arg	AR	Heterozygous	1691054	0.018	Conflicting	VUS	Autosomal Dominant Rubinstein-Taybi syndrome 2, and Menke-Hennekam syndrome 2
**53**	13	*RAI1*	c.2422G>A	p.Gly808Arg	AR	Heterozygous	196538	0.026	Tolerated	VUS	Autosomal dominant Smith-Magenis syndrome
**56**	11	*CEP290*	c.6572A>T	p.His2191Leu	AR	Heterozygous	959829	0.023	Tolerated	VUS	autosomal recessive conditions Leber congenital amaurosis 10 OMIM #611755, Bardet-Biedl syndrome 14 OMIM #615991, Joubert syndrome 5 OMIM #610188, MeckelGruber syndrome 4 OMIM #611134, and Senior-Loken syndrome 6 OMIM #610189.
**56**	11	*SDCCAG8*	c.833G>A	p.Arg278His	AR	Heterozygous	850817	0.02	Tolerated	VUS	autosomal recessive Bardet-Biedl syndrome 16 (OMIM #615993), and Senior-Loken syndrome 7 (OMIM #613615).
**56**	11	*VPS13B*	c.10939G>A	p.Ala3647Thr	AR	Heterozygous	576857	0.004	Conflicting	VUS	Cohen syndrome
**57**	13	*ADCY3*	c.1222G>A	p.Gly408Arg	AR	Heterozygous	Not listed	0.029	Conflicting	VUS	autosomal recessive severe obesity
**57**	13	*ALMS1*	c.1606C>G	p.Leu536Val	AR	Heterozygous	403950	0.059	Conflicting	VUS	Alstrom syndrome
**58**	5	*CEP290*	c.3722A>G	p.Lys1241Arg	AR	Heterozygous	Not listed	.	Conflicting	VUS	autosomal recessive conditions Leber congenital amaurosis 10 OMIM #611755, Bardet-Biedl syndrome 14 OMIM #615991, Joubert syndrome 5 OMIM #610188, MeckelGruber syndrome 4 OMIM #611134, and Senior-Loken syndrome 6 OMIM #610189.
**59**	7	*PCSK1*	c.661A>G	p.Asn221Asp	AR	Heterozygous	14040	6.6	Conflicting	RISK	obesity with impaired prohormone processing
**61**	3	*BBS1*	c.1169T>G	p.Met390Arg	AR	Heterozygous	12143	0.28	Conflicting	Pathogenic	Bardet-Biedl syndrome 1
**61**	3	*LEPR*	c.1166G>A	p.Ser389Asn	AR	Heterozygous	297992	0.0098	Damaging	VUS	autosomal recessive obesity and hypogonadotropic hypogonadism due to leptor receptor deficiency
**61**	3	*NCOA1*	c.2428G>A	p.Asp810Asn	AR	Heterozygous	Not listed	0.077	Conflicting	VUS	autosomal dominant severe obesity
**63**	15	*CEP290*	c.3722A>G	p.Asn1258Ser	AR	Heterozygous	834384	0.0085	Conflicting	VUS	autosomal recessive conditions Leber congenital amaurosis 10 OMIM #611755, Bardet-Biedl syndrome 14 OMIM #615991, Joubert syndrome 5 OMIM #610188, MeckelGruber syndrome 4 OMIM #611134, and Senior-Loken syndrome 6 OMIM #610189.
**63**	15	*ALMS1*	c.4046A>G	p.His1349Arg	AR	Heterozygous	837210	0.058	Tolerated	VUS	Alstrom syndrome
**63**	15	*RAB23*	c.481+4A>C	Intronic	AR	Heterozygous	Not listed	0.023	Not applicable	VUS	Carpenter syndrome
**63**	15	*SH2B1*	c.1846T>C	p.Ser616Pro	AR	Heterozygous	929780	0.087	Conflicting	VUS	obesity, insulin resistance and maladaptive behavior phenotypes

**64**	6	*NCOA1*	c.4127T>C	p.Leu1376Pro	AR	Heterozygous	Not listed	0.12	Conflicting	VUS	autosomal dominant severe obesity
**64**	6	*PCNT*	c.3868C>T	p.Arg1290Cys	AR	Heterozygous	Not listed	0.016	Tolerated	VUS	autosomal recessive microcephalic osteodysplastic primordial dwarfism, type II
**64**	6	*PCSK1*	c.375G>A	p.Met125Ile	AR	Heterozygous	906974	0.031	Conflicting	Pathogenic	autosomal recessive obesity with impaired prohormone processing
**64**	6	*RPGRIP1L*	c.251G>A	p.Arg84Gln	AR	Heterozygous	319668	0.062	Conflicting	VUS	COACH syndrome 3 OMIM #619113, Joubert syndrome 7 OMIM #611560, and Meckel syndrome 5 OMIM #611561.
**65**	9	*MKKS*	c.697A>C	p.Ile233Leu	AR	Heterozygous	532031	0.054	Tolerated	VUS	Bardet-Biedl syndrome OMIM #605231 and McKusick-Kaufman syndrome OMIM #236700
**65**	9	*NCOA1*	c.3111C>T	p. =	AR	Heterozygous	Not listed	0.071	Not applicable	VUS	autosomal dominant severe obesity
**65**	9	*VPS13B*	c.5528A>T	p.Glu1843Val	AR	Heterozygous	966951	0.032	Conflicting	VUS	autosomal dominant severe obesity
**66**	9	*BBS9*	c.396G>C	p.Gln132His	AR	Heterozygous	841915	0.042	Damaging	VUS	Bardet-Biedl syndrome 9
**72**	4	*BBS2*	c.1313C>G	p.Ser438Cys	AR	Heterozygous	Not listed	Not present	Conflicting	VUS	autosomal recessive Bardet-Biedl syndrome 2 OMIM #615981 and autosomal recessive retinitis pigmentosa 74 OMIM #616562
**73**	8	*VPS13B*	c.5528A>T	p.Glu1843Val	AR	Heterozygous	966951	0.032	Conflicting	VUS	Cohen syndrome
**74**	11	*SEMA3G*	c.2245_2259del	p.Gln749_Lys753del	Unknown	Heterozygous	Not listed	0.02	Not applicable	VUS	severe, early onset obesity
**75**	13	*SEMA3F*	c.1963C>A	p.Arg655Ser	Unknown	Heterozygous	Not listed	0.058	Conflicting	VUS	obesity
**79**	18	*ALMS1*	c.7285A>C	p.Ser2429Arg	AR	Heterozygous	640904	0.022	Conflicting	VUS	Alstrom syndrome
**79**	18	*SEMA3A*	c.271A>G	p.Ile91Val	AR	Heterozygous	Not listed	0.0087	Conflicting	VUS	autosomal dominant hypogonadotropic hypogonadism 16 with or without anosmia
**82**	13	*IFT172*	c.2261A>G	p.Glu745Gly	AR	Heterozygous	Not listed	0.0029	Conflicting	VUS	autosomal recessive ciliopathy-related disorders including, retinitis pigmentosa 71 OMIM #616394, short-rib thoracic dysplasia 10 with or without polydactyly OMIM #615630, and Bardet-Biedl syndrome 20 OMIM #619471
**84**	15	*BBS1*	c.1169T>G	p.Met390Arg	AR	Heterozygous	12143	0.28	Conflicting	Pathogenic	Bardet-Biedl syndrome 1
**84**	15	*PCSK1*	c.1918A>G	p.Thr640Ala	AR	Heterozygous	436278	0.45%	Tolerated	VUS	autosomal recessive obesity with impaired prohormone processing
**84**	15	*SIM1*	c.749C>T	p.Ala250Val	AR	Heterozygous	Not listed	Not present	Conflicting	VUS	autosomal dominant isolated obesity
**86**	18	*MKKS*	c.1530G>A	p.Trp510*	AR	Heterozygous	Not listed	0.0029	Not applicable	Likely Pathogenic	Bardet-Biedl syndrome (OMIM #605231) and McKusick-Kaufman syndrome (OMIM #236700)
**86**	18	*AFF4*	c.587G>A	p.Arg196Lys	AR	Heterozygous	1363791	0.026	Conflicting	VUS	CHOPS syndrome (OMIM #616368)
**86**	18	*TUB*	c.901G>A	p.Val301Ile	AR	Heterozygous	956470	0.015	Conflicting	VUS	autosomal recessive retinal dystrophy and obesity (OMIM #616188)
**88**	10	*RPGRIP1L*	c.251G>A	p.Arg84Gln	AR	Heterozygous	319668	0.062	Conflicting	VUS	autosomal recessive COACH syndrome 3 (OMIM #619113), Joubert syndrome 7 (OMIM #611560), and Meckle syndrome 5 (OMIM #611561)
**88**	*10*	*PCSK1*	c.661A>G	p.Asn221Asp	AR	Heterozygous	14040	6.6	Conflicting	RISK	autosomal recessive obesity with impaired prohormone processing (OMIM #600955), Some heterozygous variants may impart a susceptibility to obesity (OMIM #612362)
**89**	7	*NCOA1*	c.3416G>A	p.Ser1139Asn	AR	Heterozygous	Not listed	0.012	Conflicting	VUS	autosomal dominant severe obesity
**90**	5	*NRP1*	c.608G>C	p.Gly203Ala	Unknown	Heterozygous	Not listed	0.00088	Conflicting	VUS	Semaphorin 3 signaling and may be associated with obesity
**93**	11	*BBS9*	c.396G>C	p.Gln132His	AR	Heterozygous	841915	0.042	Damaging	VUS	Bardet-Biedl syndrome 9 (OMIM #615986)
**94**	13	*NRP1*	c.608G>C	p.Gly203Ala	Unknown	Heterozygous	Not listed	0.00088	Conflicting	VUS	Semaphorin 3 signaling and may be associated with obesity
**96**	11	*BBS7*	c.442A>C	p.Asn148His	AR	Heterozygous	1030351	Not present	Conflicting	VUS	Bardet-Biedl syndrome 7 (OMIM #615984)
**96**	11	*KSR2*	c.735G>A	p. =	AR	Heterozygous	Not listed	0.003	Not applicable	VUS	autosomal dominant non-syndromic obesity characterized by early-onset hyperphagia, low heart rate, reduced basal metabolic rate, and severe insulin resistance
**97**	12	*RPGRIP1L*	c.1709A>G	p.Lys570Arg	AR	Heterozygous	530893	0.02	Conflicting	VUS	autosomal recessive COACH syndrome 3 (OMIM #619113),

**99**	3	*MC4R*	c.227A>G	p.His76Arg	AR	Heterozygous	Not listed	0.008	Conflicting	VUS	Joubert syndrome 7 (OMIM #611560), and Meckle syndrome 5 (OMIM #611561) severe, early onset obesity with features including hyperphagia, increased linear growth, preserved reproductive function, and hyperinsulenemia.
**99**	3	*VPS13B*	c.7669G>A	p.Asp2557Asn	AR	Heterozygous	585720	0.04	Tolerated	VUS	Cohen syndrome (OMIM #216550)
**101**	7	*INPP5E*	c.1544G>A	p.Arg515Gln	AR	Heterozygous	1063364	0.046	Conflicting	VUS	autosomal recessive intellectual disability with truncal obesity, retinal dystorphy and micropenis (OMIM #610156)
**101**	7	*PLXNA2*	c.2896G>A	p.Glu966Lys	Unknown	Heterozygous	Not listed	0.004	Conflicting	VUS	autism spectrum disorder. Semaphorin 3 signaling and maybe associated with obesity.
**101**	7	*SEMA3F*	c.1963C>A	p.Arg655Ser	Unknown	Heterozygous	Not listed	0.058	Conflicting	VUS	obesity
**102**	7	*GNAS*	c.1091C>A	p.Ala364Asp	AR	Heterozygous	Not listed	Not present	Conflicting	VUS	autosomal dominant pseudohypoparathyroidism Ia, Ib, and Ic (OMIM #103580, #603233, and #612462), associated with short statue, obesity, skeletal abnormalities, and other features, without hormone resistance.
**104**	12	*BDNF*	c.*9T>Cq	Post-coding	AR	Heterozygous	Not listed	0.0029	Not applicable	VUS	autosomal dominant obesity with neurodevelopmental differences, while even larger deletions implicate BDNF as the cause of obesity in WAGRO syndrome. Minimal evidence implicates missense and other single nucleotide variants in BDNF as cause of autosomal dominant obesity.
**108**	9	*CREBBP*	c.2323A>G	p.Met775Val	AR	Heterozygous	1305241	Not present	Damaging	VUS	autosomal dominant Rubinstein-Taybi syndrome 1 (OMIM #180849). An allelic disorder, Menke-Hennekam syndrome 1 has been associated with variants in exons 30 or 31 of CREBBP (OMIM #618332).
**109**	8	*BBS1*	c.1169T>G	p.Met390Arg	AR	Heterozygous	12143	0.28	Conflicting	Pathogenic	Bardet-Biedl syndrome 1 (OMIM #209900)
**109**	8	*ADCY3*	c.923A>G	p.Asn308Ser	AR	Heterozygous	Not listed	Not present	Conflicting	VUS	autosomal recessive severe obesity
**109**	8	*BBS1*	c.1645G>T	p.Glu549*	AR	Heterozygous	12144	0.0087	Not applicable	Pathogenic	Bardet-Biedl syndrome 1 (OMIM #209900)
**113**	5	*ADCY3*	c.2042C>A	p.Ala681Asp	AR	Heterozygous	Not listed	0.0087	Conflicting	VUS	autosomal recessive severe obesity
**113**	5	*BBS9*	c.396G>C	p.Gln132His	AR	Heterozygous	841915	0.042	Damaging	VUS	Bardet-Biedl syndrome 9 (OMIM #615986)
**113**	5	*ISL1*	c.755A>G	p.Asn252Ser	Unknown	Heterozygous	Not listed	0.22	Conflicting	VUS	Dilated cardiomyopathy, or congentital heart defects, and with obesity
**113**	5	*SEMA3F*	c.1963C>A	p.Arg655Ser	Unknown	Heterozygous	Not listed	0.058	Conflicting	VUS	obesity
**113**	5	*TUB*	c.1421C>T	p.Thr474Met	AR	Heterozygous	1485765	0.0098	Conflicting	VUS	autosomal recessive retinal dystrophy and obesity (OMIM #616188)
**114**	5	*PCSK1*	c.1918A>G	p.Thr640Ala	AR	Heterozygous	436278	0.45	Tolerated	VUS	autosomal recessive obesity with impaired prohormone processing (OMIM #600955), Some heterozygous variants may impart a susceptibility to obesity (OMIM #612362)
**116**	10	*CFAP418*	c.518A>C	p.Lys173Thr	AR	Heterozygous	1015526	Not present	Damaging	VUS	autosomal recessive Bardet-Biedl syndrome 21 (OMIM #617406), autosomal cone-rod dystrophy 16 (OMIM #614500), and autosomal recessive retinitis pigmentosa 64 (OMIM #614500).
**117**	8	*UCP3*	c.58G>A	p.Ala20Thr	AR	Heterozygous	Not listed	0.029	Conflicting	VUS	severe obesity
**118**	6	*GNAS*	c.1337C>T	p.Ala446Val	AR	Heterozygous	Not listed	0.02	Conflicting	VUS	Maternally-inherited pathogenic variants in GNAS are associated with autosomal dominant pseudohypoparathyroidism Ia, Ib, and Ic OMIM #103580, #603233, and #612462). Paternally-inherited pathogenic variants in GNAS are associated with autosomal dominant pseudopsuedohypoparathyroidism (OMIM #612463).

**118**	6	*LEPR*	c.82A>G	p.Ile28Val	AR	Heterozygous	Not listed	0.024	Conflicting	VUS	autosomal recessive obesity and hypogonadotropic hypogonadism due to leptor receptor deficiency
**118**	6	*PLXNA1*	c.2587G>C	p.Asp863His	Unknown	Heterozygous	Not listed	0.016	Conflicting	VUS	developmental delay, and autism spectrum disorder phenotypes. Rare variants have been shown to affect Semaphorin 3 signaling and maybe associated with obesity.
**120**	3	*CEP290*	c.6893A>G	p.Gln2298Arg	AR		1039651	Not present	Tolerated	VUS	autosomal recessive Leber congenital amaurosis 10 (OMIM #611755).
**120**	3	*MKS1*	c.857A>G	p.Asp286Gly	AD	Homozygous	445724	0.099	Conflicting	VUS	autosomal recessive Bardet-Biedl syndrome 13 (OMIM #615990), autosomal recessive Meckel-Gruber syndrome 1 (OMIM #249000).
**120**	3	*MKS1*	c.83T>C	p.Val28Ala	AD	Homozygous	594505	0.007	Conflicting	VUS	autosomal recessive Bardet-Biedl syndrome 13 (OMIM #615990), autosomal recessive Meckel-Gruber syndrome 1 (OMIM #249000).
**120**	3	*SDCCAG8*	c.947T>C	p.Met316Thr	AR	Heterozygous	847765	0.076	Conflicting	VUS	autosomal recessive Bardet-Biedl syndrome 16 (OMIM #615993), and Senior-Loken syndrome 7 (OMIM #613615).
**120**	3	*VPS13B*	c.11668G>A	p.Asp3890Asn	AR	Heterozygous	1060017	0.0062	Conflicting	VUS	Cohen syndrome (OMIM #216550)
**121**	12	*BBS9*	c.396G>C	p.Gln132His	AR	Heterozygous	841915	0.042	Damaging	VUS	Autosomal recessive Bardet-Biedl syndrome 9 (OMIM #615986)
**121**	12	*SDCCAG8*	c.833G>A	p.Arg278His	AR	Heterozygous	850817	0.02	Tolerated	VUS	autosomal recessive Bardet-Biedl syndrome 16 (OMIM #615993), and Senior-Loken syndrome 7 (OMIM #613615).
**122**	5	*INPP5E*	c.892A>T	p.Asn298Tyr	AR	Heterozygous	848442	Not present	Conflicting	VUS	autosomal recessive intellectual disability with truncal obesity, retinal dystorphy and micropenis (OMIM #610156)
**122**	5	*SEMA3G*	c.640dup	p.Ser214Phefs*4	AR	Heterozygous	Not listed	0.0085	Not applicable	VUS	severe, early onset obesity
**123**	14	*KIDINS220*	c.2210A>G	p.Lys737Arg	AR, AD	Heterozygous	Not listed	Not present	Conflicting	VUS	de novo in patients with spastic paraplegia, intellectual disability, nystagmus, and obesity
**123**	14	*SDCCAG8*	c.833G>A	p.Arg278His	AR	Heterozygous	850817	0.02	Tolerated	VUS	Bardet-Biedl syndrome 9
**124**	10	*IFT172*	c.4210G>A	p.Gly140Ser	AR	Heterozygous	864375	0.0085	Conflicting	VUS	spectrum of autosomal recessive ciliopathy-related disorders including, retinitis pigmentosa 71 OMIM #616394, short-rib thoracic dysplasia 10 with or without polydactyly OMIM #615630, and Bardet-Biedl syndrome 20 OMIM #619471.
**125**	8	*CREBBP*	c.964G>A	p.Val322Met	AR	Heterozygous	Not listed	0.00088	Conflicting	VUS	autosomal dominant Rubinstein-Taybi syndrome 1 (OMIM #180849). An allelic disorder, Menke-Hennekam syndrome 1 has been associated with variants in exons 30 or 31 of CREBBP (OMIM #618332).
**126**	11	*ALMS1*	c.5438T>C	p.Val1813Ala	AR	Heterozygous	1363233	Not present	Conflicting	VUS	Alstrom syndrome (OMIM #203800)
**126**	11	*RPGRIP1L*	c.251G>A	p.Arg84Gln	AR	Heterozygous	319668	0.062	Conflicting	VUS	autosomal recessive COACH syndrome 3 (OMIM #619113), Joubert syndrome 7 (OMIM #611560), and Meckle syndrome 5 (OMIM #611561)
**126**	11	*SH2B1*	c.269C>A	p.Pro90His	AR	Heterozygous	727734	0.46	Conflicting	VUS	obesity, insulin resistance and maladaptive behavior phenotypes
**126**	11	*TUB*	c.161C>A	p.Thr54Lys	AR	Heterozygous	1006177	0.0087	Conflicting	VUS	retinal dystrophy and obesity
**127**	8	*ALMS1*	c.7234A>G	p.Ser2412Gly	AR	Heterozygous	606844	Not present	Conflicting	VUS	Alstrom syndrome (OMIM #203800)
**127**	8	*NTRK2*	c.250G>A	p.Glu84Lys	AR	Heterozygous	600456	Not present	Conflicting	VUS	autosomal dominant obesity, hyperphagia, and developmental delay (OMIM #613886).
**127**	8	*SH2B1*	c.1931C>T	p.Pro644Leu	AR	Heterozygous	608937	0.008	Conflicting	VUS	obesity, insulin resistance and maladaptive behavior phenotypes
**128**	17	*VPS13B*	c.8318T>C	p.Val2773Ala	AR	Heterozygous	Not listed	Not present	Conflicting	VUS	autosomal recessive Cohen syndrome (OMIM #216550)
**129**	5	*KSR2*	c.731C>T	p.Pro244Leu	AR	Heterozygous	Not listed	Not present	Conflicting	VUS	autosomal recessive Cohen syndrome (OMIM #216550)
**129**	5	*MKKS*	c.67A>G	p.Arg23Gly	AR	Heterozygous	497476	0.11	Conflicting	VUS	autosomal recessive Bardet-Biedl syndrome 6 (OMIM #605231)

**129**	5	*MKS1*	c.857A>G	p.Asp286Gly	AR	Heterozygous	445724	0.099	Conflicting	VUS	autosomal dominant non-syndromic obesity characterized by early-onset hyperphagia, low heart rate, reduced basal metabolic rate, and severe insulin resistance
**129**	5	*MKS1*	c.83T>C	p.Val28Ala	AR	Heterozygous	594505	0.007	Conflicting	VUS	autosomal recessive Bardet-Biedl syndrome 13 (OMIM #615990), autosomal recessive Meckel-Gruber syndrome 1 (OMIM #249000).
**129**	5	*SH2B1*	c.1028C>T	p.Thr343Met	AR	Heterozygous	Not listed	0.048	Conflicting	VUS	obesity, insulin resistance and maladaptive behavior phenotypes
**131**	6	*BBS1*	c.1645G>T	p.Glu549*	AR	Heterozygous	12144	0.0087	Not applicable	Pathogenic	autosomal recessive Bardet-Biedl syndrome 1 (OMIM #209900)
**132**	9	*POMC*	c.26C>T	p.Ser9Leu	AR, AD	Heterozygous	983342	0.0054	Tolerated	VUS	autosomal recessive obesity, adrenal insufficiency, and red hair due to POMC deficiency
**132**	9	*PCNT*	c.8947C>T	p.Leu2983Phe	AR	Heterozygous	197082	0.071	Conflicting	VUS	autosomal recessive microcephalic osteodysplastic primordial dwarfism, type II
**133**	8	*EP300*	c.376A>G	p.Met126Val	AR	Heterozygous	134042	0.02	Conflicting	VUS	Autosomal Dominant Rubinstein-Taybi syndrome 2, and Menke-Hennekam syndrome 2
**134**	8	*BBS1*	c.1595G>T	p.Arg532Leu	AR	Heterozygous	862497	0.0029	Conflicting	VUS	autosomal recessive Bardet-Biedl syndrome 1 (OMIM #209900)
**134**	8	*PLXNA3*	c.1297C>T	p.Arg433Cys	Unknown	Hemizygous	Not listed	0.01	Conflicting	VUS	obesity, mild intellectual disability, and autism spectrum disorder
**134**	8	*RAB23*	c.536A>C	p.Glu179Ala	AR	Heterozygous	357642	0.081	Conflicting	VUS	autosomal recessive Carpenter syndrome (OMIM #201000)
**134**	8	*TUB*	c.1421C>T	p.Thr474Met	AR	Heterozygous	1485765	0.0098	Conflicting	VUS	retinal dystrophy and obesity
**136**	6	*LEPR*	c.2681C>G	p.Pro894Arg	AR	Heterozygous	Not listed	0.08	Conflicting	VUS	autosomal recessive obesity and hypogonadotropic hypogonadism due to leptor receptor deficiency
**139**	13	*ADCY3*	c.2042C>A	p.Ala681Asp	AR	Heterozygous	2405251	0.0087	Conflicting	VUS	autosomal recessive severe obesity
**139**	13	*PCSK1*	c.661A>G	p.Asn221Asp	AR	Heterozygous	14040	6.6	Conflicting	RISK	autosomal recessive obesity with impaired prohormone processing (OMIM #600955), Some heterozygous variants may impart a susceptibility to obesity (OMIM #612362)
**139**	13	*PLXNA2*	c.2494T>C	p.Cys832Arg	AR	Heterozygous	Not listed	Not present	Damaging	VUS	autism spectrum disorder. Semaphorin 3 signaling and maybe associated with obesity.
**140**	15	*CEP290*	c.1623+2C>A	Splicing	AR	Heterozygous	1432036	Not present	Not applicable	Pathogenic	autosomal recessive Leber congenital amaurosis 10 (OMIM #611755).
**140**	15	*SEMA3F*	c.981G>A	p.Pro327=	AR	Heterozygous	Not listed	0.032	Not applicable	VUS	Obesity

VUS: Variant of Uncertain Significance, AR: Autosomal Recessive, AD: Autosomal Dominant, IDN: Identification number.

Table S1: Variant Analysis from Pediatric Puerto Rican Obesity Cohort.
